# Propagation of Respiratory Aerosols by the Vuvuzela

**DOI:** 10.1371/journal.pone.0020086

**Published:** 2011-05-23

**Authors:** Ka-Man Lai, Christian Bottomley, Ruth McNerney

**Affiliations:** 1 Healthy Infrastructure Research Centre, Department of Civil, Environmental and Geomatic Engineering, University College London, London, United Kingdom; 2 MRC Tropical Epidemiology Group, London School of Hygiene and Tropical Medicine, London, United Kingdom; 3 Department of Pathogen Molecular Biology, London School of Hygiene and Tropical Medicine, London, United Kingdom; New York State University at Albany, United States of America

## Abstract

Vuvuzelas, the plastic blowing horns used by sports fans, recently achieved international recognition during the FIFA World Cup soccer tournament in South Africa. We hypothesised that vuvuzelas might facilitate the generation and dissemination of respiratory aerosols. To investigate the quantity and size of aerosols emitted when the instrument is played, eight healthy volunteers were asked to blow a vuvuzela. For each individual the concentration of particles in expelled air was measured using a six channel laser particle counter and the duration of blowing and velocity of air leaving the vuvuzela were recorded. To allow comparison with other activities undertaken at sports events each individual was also asked to shout and the measurements were repeated while using a paper cone to confine the exhaled air. Triplicate measurements were taken for each individual. The mean peak particle counts were 658×10^3^ per litre for the vuvuzela and 3.7×10^3^ per litre for shouting, representing a mean log_10_ difference of 2.20 (95% CI: 2.03,2.36; p<0.001). The majority (>97%) of particles captured from either the vuvuzela or shouting were between 0.5 and 5 microns in diameter. Mean peak airflows recorded for the vuvuzela and shouting were 6.1 and 1.8 litres per second respectively. We conclude that plastic blowing horns (vuvuzelas) have the capacity to propel extremely large numbers of aerosols into the atmosphere of a size able to penetrate the lower lung. Some respiratory pathogens are spread via contaminated aerosols emitted by infected persons. Further investigation is required to assess the potential of the vuvuzela to contribute to the transmission of aerosol borne diseases. We recommend, as a precautionary measure, that people with respiratory infections should be advised not to blow their vuvuzela in enclosed spaces and where there is a risk of infecting others.

## Introduction

Aerosols play an important role in the spread of communicable diseases [1], [2]. Aerosol transmission can be airborne, where contaminated droplet nuclei exhaled by an infected individual are inhaled by a susceptible individual. A second route of infection is when deposited droplets are carried to the mouth or nose through physical contact, often by hand [3]. Airborne aerosol transmission is believed to make a major contribution to the spread of diseases such as tuberculosis and measles [4], [5]. Aerosols have also been implicated in the transmission of diseases such as the common cold, chickenpox, rubella, influenza, pneumococcal disease and severe acute respiratory syndrome (SARS) [3], [6], [7], [8], [9], [10], although their contribution is less clear cut as non aerosolized respiratory secretions also contribute to the spread of these diseases. Some airborne pathogens are extremely contagious; in the USA an outbreak of measles was traced to a sporting event where transmission was found to have occurred between an athlete in the arena and spectators in the stadium, with no evidence of close contact [11]. Spread of respiratory disease is of particular concern in large crowds and at international gatherings [12], [13]. This includes the annual Muslim pilgrimage to Mecca and associated sites, during which respiratory infections are the most common cause of hospitalization [14]. The reported infections include tuberculosis and influenza and the Saudi ministry of health recommends wearing of protective face masks by those attending the Hajj [13], [15]. The emergence of epidemic strains of flu have also caused concern; in 2009 fears around the spread of influenza H1N1 resulted in a temporary ban on public events in some countries [16].

Aerosols are created and expelled into the atmosphere during coughing, sneezing, singing or talking. If the person has a respiratory infection a proportion of the aerosols may carry pathogenic particles [17], [18], [19]. The size of a contaminated aerosol droplet is crucial in determining its ability to transmit disease. Whereas large drops (>100 microns diameter) will rapidly fall to the ground smaller droplets may remain suspended in the air where evaporation can occur resulting in the formation of tiny ‘droplet nuclei’ that can stay airborne for hours or days [20], [21]. These particles can be breathed in by susceptible individuals who may then become infected. The fate of the droplet nuclei on inhalation also depends on their size; particles greater than five microns are likely to remain in the upper airways but smaller particles are more likely to deposit in the alveoli and so may transmit infections of the lower respiratory tract such as tuberculosis [22], [23].

The vuvuzela is a plastic blowing horn that has been adopted by sports fans to provide audible support for their team. It is used in several countries in Asia and Africa and is particularly popular in South Africa where it figured prominently during the recent Fédération Internationale de Football Association (FIFA) World Cup. The instrument is typically 60 cm in length, tapering from a bell end 11.5 cm in diameter to a mouthpiece of 2.5 cm. Vuvuzela playing requires forceful and sustained blowing. Air from the lungs is expelled from the mouth through vibrating lips held against the plastic mouthpiece and out through the instrument. We speculated that the mode of action may facilitate the propagation and dissemination of aerosols from the respiratory tract of the person blowing the instrument. The instrument is frequently used in crowded situations and it was therefore important to determine the extent of aerosol production to assess whether blowing the vuvuzela might assist in the spread of aerosol borne diseases.

## Results

**Figure 1:**
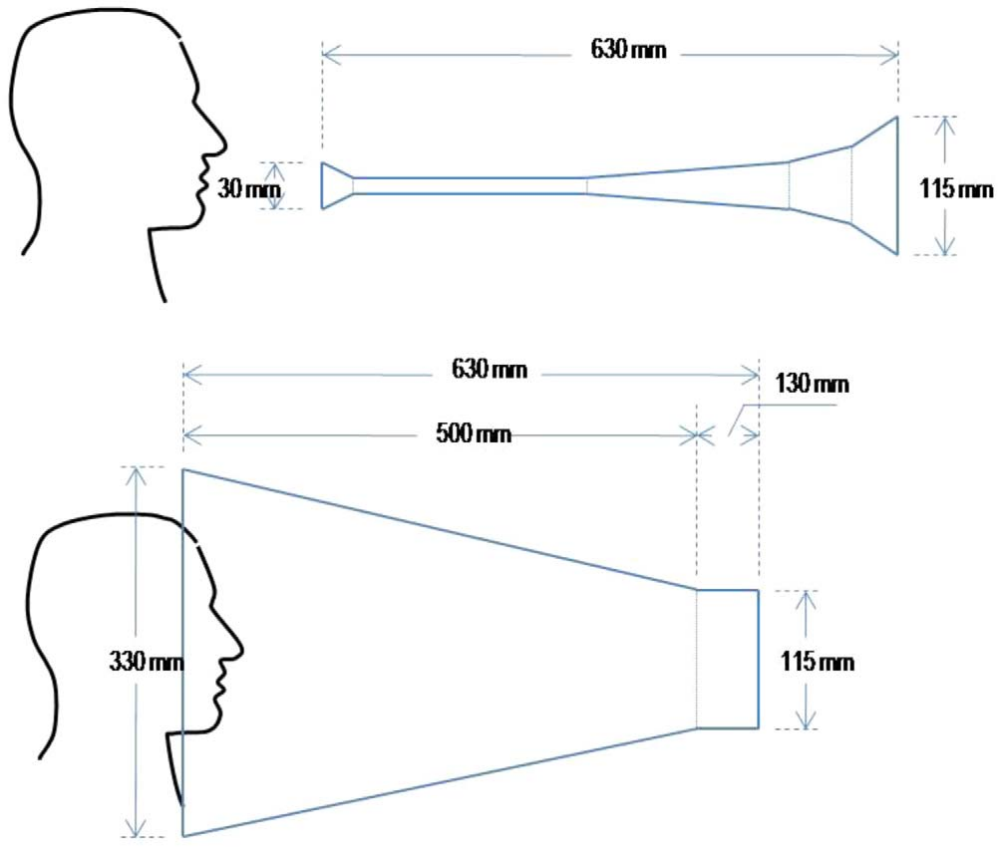
Experimental setup of vuvuzela and shouting experiments. An anemometer or particle counter were positioned at the bell of the vuvuzela to measure the velocity of air leaving the device and to capture and count aerosolized particles. Study participants also shouted into a cone tapered to the same diameter as the vuvuzela bell and measurements were repeated.

To assess the number of aerosols propagated during blowing the vuvuzela eight volunteers (4 male and 4 female) were each given an instrument and asked to blow enthusiastically, as if they were attending a football match. To enable comparison with other activities undertaken at sporting events each volunteer also shouted into a paper cone constructed to have the same 115 mm diameter exhale opening as the vuvuzela (Figure 1). Particles exiting the vuvuzela or shouting cone were assessed using a laser particle counter and enumerated in six categories according to their diameter. The velocity of air as it exited the devices was measured with a hot-wire anemometer and peak airflows were recorded. The duration of playing was recorded with a stopwatch. Triplicate experiments were undertaken for each individual tested.

### Particle counts and size distribution

Airborne particles exiting the instrument were measured every second throughout the experiment and reported as particles per litre. The mean concentration of particles recorded from playing the vuvuzela and shouting were 658×10^3^ and 3.7×10^3^ per litre respectively. To compare the number of particles emitted by an individual when shouting to the number emitted when playing the vuvuzela, the data were log_10_ transformed and the difference was calculated for each individual. The mean log_10_ difference was 2.20 (95% CI: 2.03,2.36; p<0.001). Men expelled particles at a higher mean concentration than the women when playing the vuvuzela (741×10^3^ vs 575×10^3^ per litre) although this was not statistically significant (p = 0.69).When shouting there was no difference in the numbers of particles captured (male:female; 3.4×10^3^ vs 3.9×10^3^ per litre, p = 0.89).

**Figure 2:**
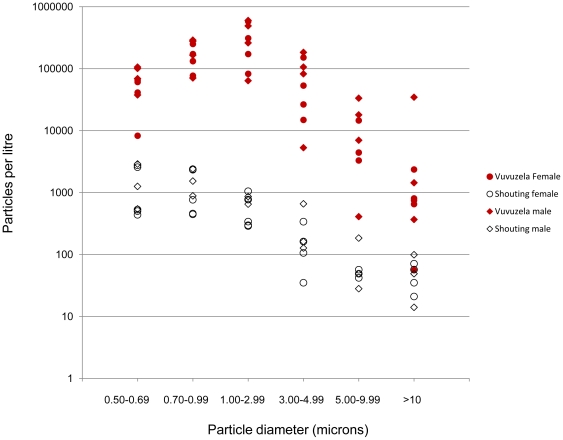
Concentration of airborne particles exiting the vuvuzela or shouting cone by their diameter. Peak concentration of particles captured at the exit of the vuvuzela and shouting cone when used by eight volunteers, four female and four male. Data points are means of triplicate experiments.

Aerosols were enumerated in six size categories according to the diameter of the particle: 0.5–0.7 µm; 0.7–1.0 µm; 1.0–3.0 µm; 3.0–5.0 µm; 5.0–10.0 µm and >10.0 µm. The distribution of particles by size category is presented in Figure 2. The great majority (97%) of particles captured from both the vuvuzela and the shouting cone were between 0.5 and 5 microns in diameter and small enough to enter the lower respiratory tract. The geometric mean (GM) particle diameter was calculated for each experiment and is presented in Table 1. Slightly larger particles were emitted when playing the vuvuzela compared to shouting (the mean difference in GM diameter was 0.34 µ (95% CI: 0.11,0.57; p = 0.01).

**Table 1:** Exhale duration, peak air velocity, particle concentration and mean particle diameter recorded during playing the vuvuzela and shouting by four male and four female volunteers.

ID	Experiment	Duration	Peak velocity	Peak particle conc.	Average particle conc.	GM§ Diameter
		s	m/s	1,000's per L	1,000's per L	microns
F1	Shout	2.4(0.3)	0.2(0.1)	6.1(0.6)	6.1(0.4)	0.8(0.0)
	Vuvu	1.5(0.2)	0.7(0.3)	606(223)	351(74)	1.3(0.1)
F2	Shout	3.9(0.7)	0.1(0.0)	6.5(0.5)	6.4(0.3)	0.9(0.0)
	Vuvu	3.1(0.9)	0.4(0.1)	1077(109)	680(44)	1.5(0.1)
F3	Shout	1.8(0.7)	0.1(0.0)	1.2(0.1)	1.3(0.1)	0.9(0.1)
	Vuvu	1.4(0.1)	0.3(0.1)	220(260)	134(143)	1.2(0.1)
F4	Shout	2.4(1.2)	0.2(0.1)	1.9(0.1)	1.8(0.1)	1.0(0.1)
	Vuvu	2.3(0.4)	0.3(0.1)	396(335)	263(280)	1.2(0.1)
M1	Shout	1.7(0.6)	0.2(0.1)	6.0(0.1)	6.0(0.3)	0.8(0.0)
	Vuvu	2.4(0.5)	0.8(0.1)	1197(245)	669(235)	1.5(0.3)
M2	Shout	1.3(0.1)	0.2(0.0)	1.5(0.3)	1.5(0.2)	1.1(0.1)
	Vuvu	2.2(0.1)	0.4(0.1)	178(140)	114(81)	0.9(0.1)
M3	Shout	1.5(0.5)	0.2(0.0)	3.2(2.5)	2.9(1.8)	1.4(0.4)
	Vuvu	2.0(0.7)	0.3(0.1)	645(120)	401(185)	1.7(0.1)
M4	Shout	2.3(0.5)	0.1(0.0)	3.0(0.8)	2.9(0.7)	0.9(0.0)
	Vuvu	2.0(0.1)	1.5(0.4)	944(225)	602(181)	1.3(0.1)
**Mean** ‡	Shout	2.2(0.8)	0.2(0.1)	3.7(2.2)	3.6(2.2)	1.0(0.2)
	Vuvu	2.1(0.5)	0.6(0.4)	658(386)	402(228)	1.3(0.2)

† Results presented here are means from three repetitions (the standard deviation of the repetitions is in brackets).

‡ Mean and standard deviation of the means presented.

§ Geometric mean diameter of particles at peak concentration.

### Exhale time, velocity and volume

The mean duration for vuvuzela playing events was 2.1 sec (range: 1.25–3.90 sec) and the shouting lasted for an average of 2.2 sec (range: 0.96–4.72 sec). The peak velocity of air exiting the vuvuzelas was higher than from the shouting cone with a mean of 0.59 ms^−1^ (range:0.12–1.80 ms^−1^;) compared to a shouting mean of 0.18 ms^−1^ (range: 0.07–0.32 ms^−1^) and this difference was statistically significant (p = 0.03). This was equivalent to airflow of 6.1 and 1.8 Ls^−1^ respectively, for the vuvuzela and shouting. Although the duration in playing vuvuzelas between females and males were similar (2.1 sec), the mean peak airflow was nearly double in males compared to females, 7.9 compared to 4.3 Ls^−1^ (this difference was not statistically significant p = 0.19). The difference between females and males in shouting was not as apparent, although males also had a higher peak airflow compared to females, 2.1 compared to 1.6 Ls^−1^ (p = 0.37).

## Discussion

We have estimated the numbers of aerosols exiting the vuvuzela when blown by male and female adults. In triplicate experiments from eight individuals the mean concentration of particles exiting the vuvuzela was 658,000 per litre. The mean peak volume of air exiting the instrument was 6.1 litres per second. Thus we estimate that approximately 4 million particles per second were being disseminated from the vuvuzela at peak blowing times. For shouting we estimated a peak aerosol concentration of 3,700 per litre or 7,000 particles per second (assuming peak flow volume of 1.8 Ls^−1^). The data we obtained for shouting is in broad agreement with a recent study of particles exhaled by healthy adults during normal to deep breathing (tidal volume range: 20–80%) where between 5 and 5,000 droplets per litre were recorded [24]. The differences we observed between male and female volunteers might be explained by differences in their lung capacities, however this was not measured [24]. Our results suggest that the vuvuzela is an efficient means of propagating large numbers of aerosols. The great majority of particles measured were of a size that could remain suspended in the air as droplet nuclei and would be capable of entering the alveolar airspaces of the lung. During normal (resting) breathing an adult inhales approximately 7 litres of air each minute, of which 5 litres reaches the respiratory bronchioles [25]. When attending a sporting event and surrounded by vuvuzela players a spectator could expect to inhale large numbers of respiratory aerosols over the course of the event. Actual exposure would be affected by the proximity of the vuvuzelas and ambient ventilation which would serve to dilute the stream of particles.

The large number of aerosols emitted by the vuvuzela raises the possibility that, if used by persons with an infection of the respiratory tract, they could act a conduit for the spread of infectious particles. For ethical and safety reasons we only examined healthy volunteers during this study; assessment of pathogenicity of aerosols disseminated by the vuvuzela will require further study using patients with known respiratory infections. Aerosols can be created at various locations within the respiratory tract [26] and carriage of pathogens by exhaled aerosols depends on the site of infection and the quantity pathogenic particles in the airways [18].

We speculate that aerosols propagated while blowing the vuvuzela may originate in either the lower or upper respiratory tract, or the mouth. To obtain the desired trumpet sound when blowing the vuvuzela air is forced through the lips into the opening of the instrument which may serve to create further aerosols, or alter the size of droplets produced elsewhere in the respiratory tract. In addition to the manner in which the instrument was blown the number of contaminated particles expelled will vary according to the pathogen, the site of infection and the extent of disease. Some infections may result in inflammation and physiological changes within the respiratory tract that would affect the person's capacity to blow the vuvuzela [27]. In addition, some conditions are associated with changes in the rheology of respiratory secretions that might affect aerosol formation [28], [29]. Studies of cough aerosols from pulmonary tuberculosis patients and cystic fibrosis patients with bacterial infections found that the concentration of infectious particles varied widely between patients [30], [31]. To attain an accurate assessment of the vuvuzela's potential to disseminate infected aerosols, sample sizes will need to be increased to include individuals having a range of upper and lower respiratory tract infections. Symptomatic and non symptomatic carriers should be assessed. In addition to counting the number and size of particles, the viability of infectious particles should also be assessed. For bacterial infections this might be achieved by modification of a cough aerosol sampling system previously used to assess tuberculosis patients [30].

Coughing, sneezing, singing and talking can all produce aerosols capable of transmitting airborne respiratory diseases [17], [18], [30], [32]. Reports from earlier investigators suggest that coughs may produce up to 5,000 droplet nuclei and a sneeze may generate as many as 900,000 particles [21], [33]. The data we present suggests that blowing the vuvuzela for even a short time period has the potential to create more droplet particles than either coughing or sneezing.

There were some limitations to this study that may have had an impact on the results. The particle counter used to assess the concentration of particles recorded measurements at one second intervals and it is possible that the peak values recorded were not the maximum level of particle produced. As it was not possible to assess variation in flow rates over the blowing period the total number of particles expelled during a blowing or shouting event could not be estimated. The performance of individuals and production of aerosols may have been influenced by their respective lung capacities [24], this factor was not assessed in the experiment. The use of a paper cone to assess the droplets from shouting was not ideal as the surface areas and shape of the paper cone may increase the chance that particles attach to the surface rather than remain in the airstream, affecting the number and size of particles reaching the counter. As exhaled air cools and mixes with ambient air condensation droplets may form. Although ambient air temperature and humidity remained similar in all experiments, the difference in shape between the cone and the vuvuzela may have affected the mixing and rate of formation of these transient droplets. A further consideration is that only healthy individuals were recruited for this study, and as described above, it is possible that people with respiratory illness with impaired lung function would perform differently when blowing the vuvuzela. Nonetheless we have demonstrated that these plastic trumpets provide an excellent means of propagating respiratory aerosols, exceeding both sneezing and coughing as a means of disseminating droplet nuclei and we conclude that their potential to spread respiratory diseases requires further investigation. The frequency, duration, and vigor of vuvuzela playing will vary considerably from person to person, depending on the occasion and their expertise at blowing and we are unable to comment on the number of aerosols produced during an entire sporting event. A further factor is the environment in which they are used; open situations with a strong draft or breeze will serve to rapidly dilute the aerosols produced but transmission risks may be considerably higher in enclosed arenas. A further risk factor for disease transmission will be the density of vuvuzela players and the prevalence of respiratory infections in the population.

As far as we are aware this is the first report in the scientific press regarding the issue of aerosol dissemination by the vuvuzela and no epidemiological data regarding impact of the instrument on disease transmission have been reported. Similarly there have been no reports of disease transmission from sharing vuvuzelas, or from transfer of non aerosolized respiratory secretions that collect inside the instruments. The vuvuzela has become popular in South Africa, a country with the highest urban prevalence of tuberculosis in the world and that recently experienced a measles epidemic [34]. It has been used at domestic soccer games for the past decade and was adopted by many visiting fans during the 2010 FIFA World Cup competition. The tournament was held during late June and early July and coincided with the annual flu season. Surveillance reports show an increase in the proportion of influenza B compared to previous years, but evidence to link this to the presence of visiting spectators is not presented [35]. Similarly a number of measles cases were confirmed amongst visitors from other countries but evidence as to the source of their infections is not available [36]. The plastic vuvuzela is believed to have emerged as a child's toy, before being adopted by sports fans in Africa and parts of Asia, where it is a multi-million dollar industry. In Africa it has become a symbol of the soccer industry but vuvuzelas are also blown by fans of cricket and rugby football. They have been banned from a number of sports grounds due to the volume of noise emitted and safety concerns arising from their ability to nullify public address systems. Studies have previously suggested that vuvuzela playing poses a risk of noise induced hearing loss [37], [38]. We recommend that consideration is taken of their propensity to disseminate respiratory aerosols and that persons with respiratory infections be advised not to blow their vuvuzela in places where they risk infecting others. This should include enclosed spaces and crowded venues such as large sporting events. We also recommend that research be commissioned to determine the risks to public health posed by the vuvuzela.

## Materials and Methods

This study was undertaken at the Healthy Infrastructure Research Centre at University College London. Ethical approval was obtained from University College London Research Ethics Committee and informed consent was obtained in writing from all participants. Eight healthy volunteers, 4 males and 4 females working in the Research Centre participated. The experiments were conducted in a closed room free from drafts. The study subjects were in the age range 20 to 45 years and all self reported as being free from illness. To avoid cross contamination a new vuvuzela instrument was provided for each participant (Boogie Blast Co, Johannesburg, SA). The velocity of air leaving the instrument was measured using a hot-wire anemometer (Testo, UKFlow) and duration of playing recorded with a stopwatch. Our initial measurements showed that the average time of playing the vuvuzela was about 2 sec. To enable comparison with other activities undertaken at sporting events each individual tested was requested to also shout into a paper cone constructed to have the same diameter exhale opening as the vuvuzela (Figure 1). Subjects were requested to hold their shout for about 2 sec (not compulsory) and to shout the word “GO”.

Particles exiting the vuvuzela or the shouting cone were measured using a six channel laser particle counter (Lighthouse 5016, UK). Particles were enumerated in six categories according to their diameter: 0.5–0.7 µm; 0.7–1.0 µm; 1.0–3.0 µm; 3.0–5.0 µm; 5.0–10.0 µm and >10.0 µm. A 0.047 litre sample of air was tested every second and the number of particles recorded. Analyses are based on either the average or peak concentration observed during the vuvuzela or shouting event. Triplicate experiments were undertaken for each individual tested. Volume of the airflow was estimated by multiplying the peak air velocity recorded in the anemometer with the duration of playing and shouting and the surface area of the exhale opening of the vuvuzela and paper cone.

### Statistical analysis

In each experiment (in which an individual either shouted or blew on a vuvuzela) data were collected on particle concentration every second. These data were summarized in one of two ways: i) as the concentration observed in the 2^nd^ second after the start of the experiment, which usually corresponded to the peak concentration or ii) as the average concentration over the length of the shout or vuvuzela blow. All analyses were carried out using both peak and average concentrations. However, since both yielded similar results, we restrict our presentation to the analysis of peak concentrations.

The geometric mean size (GM) of particles was calculated for each experiment by fitting a log normal distribution to the particle size data at peak concentration. Estimates were obtained by maximum likelihood, allowing for interval-censoring (particle sizes were recorded using the categories 0.5–0.7 µm; 0.7–1.0 µm; 1.0–3.0 µm; 3.0–5.0 µm; 5.0 – 10.0 µm and >10.0 µm).

Each individual shouted and blew the vuvuzela three times. Statistical analysis of particle concentrations and GM particle size were based on the means of these triplicate measurements; this was done to eliminate dependence in the data arising from repeat measurements on the same individual.

To compare the number of particles emitted when shouting to the number emitted when playing the vuvuzela, we log-transformed each individual's average concentration (averaged over the three measurements) when shouting and when playing the vuvuzela and calculated the difference between these log-transformed values. The confidence interval for the mean difference and p-value were based on a paired (one-sample) t-test. Differences in the average (GM) particle size between vuvuzela and shouting were similarly assessed using a paired t-test (although the data were not log-transformed in this instance).

Comparisons between men and women of particle concentration and airflow were made using permutation tests (based on the Wicoxon rank sum statistic) rather than t-tests owing to the small numbers (4 men and 4 women).

## References

[1] Duguid JP (1947). The mechanism of transmission of pathogenic organisms affecting the respiratory tract.. Rep Dep Health Scotl.

[2] Tang JW, Li Y, Eames I, Chan PK, Ridgway GL (2006). Factors involved in the aerosol transmission of infection and control of ventilation in healthcare premises.. J Hosp Infect.

[3] Hendley JO, Gwaltney JM (1988). Mechanisms of transmission of rhinovirus infections.. Epidemiol Rev.

[4] Biellik RJ, Clements CJ (1997). Strategies for minimizing nosocomial measles transmission.. Bull World Health Organ.

[5] Riley RL, Mills CC, Nyka W, Weinstock N, Storey PB (1995). Aerial dissemination of pulmonary tuberculosis. A two-year study of contagion in a tuberculosis ward. 1959.. Am J Epidemiol.

[6] Knight V (1980). Viruses as agents of airborne contagion.. Ann N Y Acad Sci.

[7] Roy CJ, Milton DK (2004). Airborne transmission of communicable infection–the elusive pathway.. N Engl J Med.

[8] Tellier R (2006). Review of aerosol transmission of influenza A virus.. Emerg Infect Dis.

[9] Hoge CW, Reichler MR, Dominguez EA, Bremer JC, Mastro TD (1994). An epidemic of pneumococcal disease in an overcrowded, inadequately ventilated jail.. N Engl J Med.

[10] Leclair JM, Zaia JA, Levin MJ, Congdon RG, Goldmann DA (1980). Airborne transmission of chickenpox in a hospital.. N Engl J Med.

[11] Ehresmann KR, Hedberg CW, Grimm MB, Norton CA, MacDonald KL (1995). An outbreak of measles at an international sporting event with airborne transmission in a domed stadium.. J Infect Dis.

[12] Rashid H, Haworth E, Shafi S, Memish ZA, Booy R (2008). Pandemic influenza: mass gatherings and mass infection.. Lancet Infect Dis.

[13] Ahmed QA, Arabi YM, Memish ZA (2006). Health risks at the Hajj.. Lancet.

[14] Al-Ghamdi SM, Akbar HO, Qari YA, Fathaldin OA, Al-Rashed RS (2003). Pattern of admission to hospitals during muslim pilgrimage (Hajj).. Saudi Med J.

[15] El-Sheikh SM, El-Assouli SM, Mohammed KA, Albar M (1998). Bacteria and viruses that cause respiratory tract infections during the pilgrimage (Haj) season in Makkah, Saudi Arabia.. Trop Med Int Health.

[16] Moloney A (2009). Questions raised over response to influenza A outbreak.. Lancet.

[17] Loudon RG, Roberts RM (1968). Singing and the dissemination of tuberculosis.. Am Rev Respir Dis.

[18] Duguid JP (1946). Expulsion of Pathogenic Organisms from Respiratory Tract.. Br Med J.

[19] Riley RL (1974). Airborne infection.. Am J Med.

[20] Wells WF (1934). On Air-Borne Infection Study II. Droplets and droplet nuclei.. American Journal of Hygiene.

[21] Duguid JP (1946). The size and the duration of air-carriage of respiratory droplets and droplet-nuclei.. J Hyg (Lond).

[22] Hatch TF (1961). Distribution and deposition of inhaled particles in respiratory tract.. Bacteriol Rev.

[23] Wells WF, Ratcliffe HL, Crumb C (1948). On the mechanics of droplet nuclei infection. II Quantitative experimental air-borne tuberculosis in rabbits.. Am J Hyg.

[24] Schwarz K, Biller H, Windt H, Koch W, Hohlfeld JM (2010). Characterization of exhaled particles from the healthy human lung–a systematic analysis in relation to pulmonary function variables.. J Aerosol Med Pulm Drug Deliv.

[25] Roberts F (2000). Respiratory Physiology Upate in Anaethesia: World Federation of Societies of Anaesthesiologists.

[26] Morawska L, Johnson GR, Ristovski ZD, Hargreaves M, Mengersen K (2009). Size distribution and sites of origin of droplets expelled from the human respiratory tract during expiratory activities.. Journal of Aerosol Science.

[27] Hall WJ, Douglas RG (1980). Pulmonary function during and after common respiratory infections.. Annu Rev Med.

[28] Serisier DJ, Carroll MP, Shute JK, Young SA (2009). Macrorheology of cystic fibrosis, chronic obstructive pulmonary disease & normal sputum.. Respir Res.

[29] Fiegel J, Clarke R, Edwards DA (2006). Airborne infectious disease and the suppression of pulmonary bioaerosols.. Drug Discov Today.

[30] Fennelly KP, Martyny JW, Fulton KE, Orme IM, Cave DM (2004). Cough-generated aerosols of Mycobacterium tuberculosis: a new method to study infectiousness.. Am J Respir Crit Care Med.

[31] Wainwright CE, France MW, O'Rourke P, Anuj S, Kidd TJ (2009). Cough-generated aerosols of Pseudomonas aeruginosa and other Gram-negative bacteria from patients with cystic fibrosis.. Thorax.

[32] Bates JH, Potts WE, Lewis M (1965). Epidemiology of Primary Tuberculosis in an Industrial School.. N Engl J Med.

[33] Chao CYH, Wan MP, Morawska L, Johnson GR, Ristovski ZD (2009). Characterization of expiration air jets and droplet size distributions immediately at the mouth opening.. Journal of Aerosol Science.

[34] World Health Organisation (2010). Global tuberculosis control:WHO Report 2010..

[35] National Institute for Communicable Diseases (2011). Severe Acute Respiratory Illness (SARI) Surveillance: Influenza Report..

[36] Blumberg L (2010). 2010 FIFA Soccer Would Cup, South Africa: Communicable Disease Risks and Surveillance..

[37] Swanepoel de W, Hall JW, Koekemoer D (2010). Vuvuzela sound measurements.. S Afr Med J.

[38] Swanepoel de W, Hall JW, Koekemoer D (2010). Vuvuzela - good for your team, bad for your ears.. S Afr Med J.

